# Gender based differences in clinical and Angiographic characteristics and outcomes of Acute Coronary Syndrome (ACS) in Asian population

**DOI:** 10.12669/pjms.35.5.743

**Published:** 2019

**Authors:** Afrasyab Altaf, Hammad Shah, Momin Salahuddin

**Affiliations:** 1 Afrasyab Altaf, (MD, PhD, FRCP), Department of Cardiology, Rehman Institute of Medical Sciences, Peshawar, Pakistan; 2 Hammad Shah, (MBBS), Department of Cardiology, Rehman Institute of Medical Sciences, Peshawar, Pakistan; 3 Momin Salahuddin, (MRCP, FRCP), Department of Cardiology, Rehman Institute of Medical Sciences, Peshawar, Pakistan

**Keywords:** Acute Coronary Syndrome, Angiographic characteristics, Clinical Outcomes, Gender

## Abstract

**Objective:**

There is very limited data about gender based differences in Acute Coronary Syndrome (ACS) in Asian population. This study was therefore aimed to ascertain gender based differences in clinical and angiographic characteristics and clinical outcomes in patient admitted with ACS.

**Methods:**

This was a cross sectional, observational study including patients admitted with diagnosis of ACS. Patients were divided into two groups (Males and Females) and their clinical characteristics were noted. Gender based assessment was done following coronary angiography.

**Results:**

A total of 386 patients were included, with 210 males and 176 females. Anterior wall myocardial infarction (AWMI) was present in 177(45.86%) patients. Mean age was 72.8±12.9 years in females and 66.8±11.2 years in males. Diabetes was present in 38.1% females and 31% males. Patients from rural population were 225(58.3%), while from urban 161(41.7%). Mean ejection fraction was 43.9±7.9% in women and 38.2±8.68% in males.

**Conclusion:**

ACS was more common in males, rural population and AWMI was most common cause. Women were more elderly and had more adverse events as compared to males. Impaired renal dynamics were more commonly observed in males. Women were less aggressively treated with coronary interventions and radial approach was better tolerated regardless of gender.

## INTRODUCTION

Acute Coronary Syndrome (ACS) is characterized by decreased perfusion of heart muscles, which includes unstable angina and myocardial infarction.^1^ Myocardial infarction is diagnosed by rise or fall of cardiac biomarkers, with addition of any one of other classic features like ischemic symptoms, dynamic ECG changes, new onset LBBB, evidence of loss of viable myocardium, appearance of new regional wall motion abnormality or presence of intracoronary thrombus.^2^

Rise in cardiac biomarkers predict severity of coronary stenosis^3^ but another study showed severe stenosis was not predictor of myocardial infarction and ACS occurred with mild to moderate stenosis in majority of patients.^4^ Vulnerable plaque rupture predict the occurrence of ACS.^5^ According to pathological studies TCFA(Thin Cap Fibro Atheroma) is main type of vulnerable plaque susceptible to rupture.^6^ Actual frequency of plaque rupture is low and doesn’t exhibit clinically, therefore additional factors including increased blood vulnerability has been hypothesized to contribute to clinical manifestation of ACS besides vulnerable plaque rupture.^7^

Age is important factor determining outcome of ACS and elderly patients have very high in hospital mortality because they are given palliative treatment mostly^8^ and do not get optimum recommended treatment.^9^ Similarly gender also affects ACS outcome but we have conflicting contradictory evidence. Increased incidence of adverse events was observed in women undergoing early invasive strategy^10^, but overall long term survival was better in woman as compared to men.^11^

There is very limited data about gender differences in clinical features, angiographic characteristics and ACS outcomes in Asian population. This study was therefore aimed to ascertain gender based differences in clinical features, angiographic characteristics and outcome of Asian patients admitted with ACS presenting to a tertiary care hospital.

## METHODS

This was a cross sectional, descriptive, observation study carried out at cardiology department of Rehman Medical Institute from 01^st^ Jan, 2018 to 30^th^ Jun 2018 a period of six months. A total of 386 patients were included in the study population using universal sampling technique. All those patients were included who were admitted or discharged with diagnosis of acute coronary syndrome fulfilling the criteria of either unstable angina or myocardial infarction.^2^ Patients who were admitted due to non-cardiac causes like severe pneumonia, ARDS, and renal failure were excluded from the study population. Data were collected using a printed questionnaire. Informed written consent was obtained and confidentiality of the patients was ensured.

Mechanical Complications included ventricular septal defect, ischemic mitral regurgitation, pericardial effusion and free wall rupture confirmed on echocardiography. Heart failure was defined according to NYHA (New York Heart Association) classification. Bundle branch block was diagnosed as presence of new onset right or left bundle branch block on ECG. Arrhythmias were broadly termed for all the supraventricular and ventricular electrical abnormalities documented on ECG. Renal dysfunction was worsening of serum creatinine more than double of the baseline during 24 hours. Left Ventricular Ejection Fraction was assessed using Left Ventricular End Diastolic Dimensions and Left Ventricular End Systolic Dimensions by modified Simpson’s method.^12^

This study was approved by research evaluation unit of Rehman Medical Institute after scrutiny of synopsis and abided by the declaration of Helsinki.

### Data Analysis

Data was analyzed by SPSS 20. The Shapiro-Wilk test was applied to check the distribution of data. Mean ± Standard Deviation was determined for quantitative variables. Qualitative variables were expressed as frequencies and percentages. Chi Square test and Fischer exact test was used to assess the association between qualitative variables and gender. Independent T-test was applied to analyze gender association with quantitative variable. *P*-value of less than 0.05 was considered as significant.

## RESULTS

A total of 386 patients were admitted with diagnoses of ACS with 210 (54.4%) males and 176 (45.6%) females. Among total patients, 177 (45.86%) patients had anterior wall myocardial infarction, 84 (21.8%) patients had inferior myocardial infarction, 38 (9.8%) patients had NSTEMI and 19 (4.9%) patients had unstable angina. Mean hospital stay was 2.8±1.8 days. Diabetes was more commonly observed in females as compared to male patients. A total of 225 (58.3%) were from rural population and 161 (41.7%) were from urban (Table-I).

**Table I:** Baseline characteristics of study group.

Variable	Male (n=210)	Female (n = 176)	P-value
Age(Years)			
Body Mass Index(kg/m^2^)	66.8±11.2	72.8±12.9	
Population	24.8	25.8	
Rural	126(60%)	99(56.3%)	
Urban	84(40%)	77(43.8%)	
Diabetes	65(31%)	67(38.1%)	
Hypertension	88(41.9%)	72(40.9%)	
Hyperlipidemia	47(22.3%)	32(18.2%)	
Smoking	101(48.1%)	5(0.3%)	0.046
Ejection Fraction	38.2±8.68	43.9±7.9	0.071
NYHA Class	2.9±1.2	3.1±1.6	0.066
Hospital Stay(days)	2.4±1.5	3.1±1.9	0.077
Heart Failure	36(17.1%)	32(18.2%)	0.024
In hospital Treatment			0.088
Aspirin	197(93.8%)	159(90.3%)	0.066
Clopidogrel	188(89.5%)	162(92%)	<0.01
Beta Blockers	174(82.8%)	141(80.1%)	0.044
CCBs	17(8.1%)	11(6.2%)	0.063
Digoxin	30(14.3%)	20(11.4%)	0.061
Ivabradine	80(38.1%)	58((32.9%)	0.091
ACEI	166(79%)	134(76.1%)	
ARBs	132(62.8%)	105(59.6%)	
Statins	185(88.1%)	144(81.6%)	
Ionotropes	84(40%)	58(32.9%)	
Enoxaparin	193(91.9%)	157(89.2%)	
Heparin	16(7.6%)	9(5.1%)	
Tirofiban	9(4.3%)	11(6.2%)	
Rivaroxeban	12(5.7%)	8(4.5%)	

**Fig.1:**
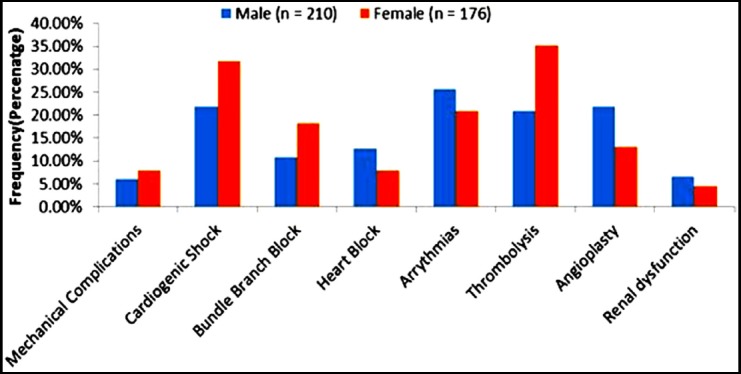
ACS outcomes in male and female patients.

Male patients had more adverse renal dynamics then female patients and had a greater propensity of heart blocks. However, overall adverse events were more frequently observed in women as compared to men leading to increase in mean hospital stay among women patients as compared to men. Gender based details of ACS outcomes are shown in Fig.1.

Coronary interventions were carried out in 186 (48.1%) patients of ACS admitted to RMI among whom 120 (31%) were males and 66 (17.1%) were females. Radial approach was better tolerated and had less hematological adverse events. More than half of the patients 100 (53.1%) were suffering from triple vessel coronary artery disease and LAD 154 (81.9%) was the most common coronary artery affected followed by Left circumflex 138 (73.4%) and right coronary artery 118 (62.8%). Left main stem was involved in 26 (13.8%) of the patients as shown in Table-II.

**Table II:** Coronary Angiography Characteristics and Procedure Outcomes in ACS.

Variable	Male (n=120)	Female (n =66)	P-value
***Coronary Angiography***			
Radial Approach	79(65.8%)	46(69.7%)	0.063
Femoral Approach	41(34.2%)	20(30.3%)	0.071
SVCAD	25(20.8%)	15(22.7%)	0.083
DVCAD	30(25%)	18(27.3%)	0.061
TVCAD	63(52.5%)	37(56.1%)	0.058
LMS Disease	17(14.2%)	9(13.6%)	0.066
LAD Disease	98(81.7%)	56(84.8%)	0.081
RCA Disease	76(63.3%)	42(63.6%)	0.099
Circumflex Disease	88(73.3%)	50(75.8%)	0.061
Single vessel PCI	68(54.2%)	29(43.9%)	0.033
Multi Vessel PCI	57(47.5%)	28(42.5%)	0.041
POBA Only	2(1.7%)	2(3%)	0.089
Hematoma Formation	6(5%)	4(6%)	0.073
Blood Transfusions	1(0.8%)	1(1.5%)	0.066

## DISCUSSION

Adequate evidence exists about age influencing ACS outcomes, but there are contradictory reports about gender effects on ACS outcomes globally. Some studies predict higher in hospital mortality for female patients,^13^ some report more adverse events in elder female patients,^8^ while others advocate no difference in mortality and better long-term survival of women patients.^11^ No data exists about epidemiology of Asian population gender effects on clinical features, angiographic characteristics and outcomes of ACS patients. This study was therefore aimed to provide subjective evidence of gender based differences in clinical features, angiographic characteristics and outcomes of Asian patients admitted with ACS presenting to a tertiary care hospital.

In a prospective cohort study involving 2,135 subjects, transition from gradual to rapid increase in incidence of ACS occurred earlier in men (51-55 year) as compared to women (56-60yr).^14^ Men had increased overall incidence of ACS than women (24.1% vs. 17.0%) and had higher incidence in corresponding age groups aswell.^14^ Our results showed preponderance of males, among patients admitted with ACS and women were more elderly at presentation as compared to men. Late increase in slow to rapid transition in women provides reasonable explanation to higher mean age of women than men observed in our results among patients of ACS.

Our results showed that majority of ACS patients were from rural population as compared to urban. Similar comparable findings were observed in Pakistani subjects where 63.2% were from rural area and 36.8% from urban. Prevalence of hyperhomocystenemia was documented as higher in northern population and male patients,^15^ and hyperhomocystenemia has positive correlation with coronary artery disease.^16^ Moreover, majority of our population lives in rural area.^17^ All these factors explain our results of raised incidence of ACS among subjects from rural areas and preponderance of male patients among total patients admitted with diagnosis of ACS.

Anterior wall myocardial infarction was most common cause of ACS accounting for 45.8% cases in our study population. Our results were consistent with other international studies which reported anterior wall myocardial infarction as leading cause of STEMI accounting for 55% cases.^18^ The difference in frequencies is due to different population selection. Our study population included all ACS patients having both STEMI and NSTEMI. Rajhans R et al, studied only 50 patients with STEMI leading to higher overall percentages in their results.^18^

Incidence of diabetes is reported to be more in females internationally^11^ and similar trend was observed in our population where majority of patients with diabetes mellitus were females (38.1% against 31% with p = 0.024). Women had more mean ejection fraction of 43.9 ±7.9% against 38.2±8.68% in males(p value = 0.044) because women have increased prevalence of heart failure with preserved ejection fraction.^19^ Another study reports male patients to have more right ventricular mass and right ventricular volume but lower ejection fraction then females due to sex hormones even in non-heart failure patients.^20^ These factors might have influenced the mean difference in ejection fraction observed in our study population.

Men had adverse renal dynamics than females in our subjects because women were less frequently exposed to interventions. Majority of our subjects have underlying renal dysfunction.^21^ Coronary interventions affects renal dynamics further by directly inducing contrast nephropathy and secondarily causing decreased renal perfusion due to more blood loss during coronary interventions. Radial approach was better tolerated because it had less frequent hematologic side effects than femoral approach.

Our results showed that apart from better renal dynamics women generally had more adverse events as compared to men because women were elder at presentation and they had greater comorbidity like diabetes, as compared to male patients. Different international studies support our results. Udell JA et al.^22^ reported increased number of overall adverse events in women as compared to male patients.

Heart failure is a poor prognostic factor after ACS and its incidence increases steeply with age.^23^ Although Torabi A et al., results showed adverse prognostic value of heart failure development after ACS but they failed to show actual disease burden in community. Our results showed 18.2% incidence of heart failure in women against 17.1% in male patients with p value of 0.66 showing no significant gender associated difference.^23^

Arrhythmias and cardiogenic shock were more frequently reported in female patients as compared to male patients. Females had less frequently undergone coronary interventions and more thrombolysis than their male counterparts. Streptokinase is the most common thrombolytic used in underdeveloped countries and arrhythmias along with hypotension are its well-recognized most common side effects.^24^ This explains the higher incidence of shock and arrhythmias in female patients of our study population.

### Limitations

Having been a cross sectional single centered study, the trends in general population cannot be truly predicted from our results, but it will serve as a platform for further population based studies.

## CONCLUSION

Incidence of ACS in Asian population is more in males, rural population and AWMI was its most common cause. Women were generally elder at presentation and suffer more frequently from adverse events. Impaired renal side effects and heart blocks were more common in male patients. Women were less aggressively treated with coronary interventions and radial approach was better tolerated in both genders.

### Authors’ Contribution

**AA, HS,** conceived, designed and did statistical analysis & editing of manuscript.

**HS, MS,** did data collection and manuscript writing.

**AA**, takes the responsibility and is accountable for all aspects of the work in ensuring that questions related to the accuracy or integrity of any part of the work are appropriately investigated and resolved.

## References

[1] Fanaroff AC, Rymer JA, Goldstein SA (2015). Acute Coronary Syndrome. JAMA.

[2] Thygesen K, Alpert JS, Jaffe AS, Simoons ML, Chaitman BR, White HD (2012). Third universal definition of myocardial infarction. Circulation.

[3] Ayesha Saleem A, Ali A (2017). Correlation of C-Reactive Protein and Cardiac Enzymes with Angiographic Severity of Coronary Artery Disease in Pakistani Patients with Acute Coronary Syndrome. J Coll Physicians Surg Pak.

[4] Falk E, Shah PK, Fuster V (1995). Coronary plaque disruption. Circulation.

[5] Schaar JA, Muller JE, Falk E, Virmani R, Fuster V, Serruys PW (2004). Terminology for high-risk and vulnerable coronary artery plaques:Report of a meeting on the vulnerable plaque June 17 and 18 2003, Santorini, Greece. Eur Heart J.

[6] Burke AP, Farb A, Malcom GT, Liang YH, Smialek J, Virmani R (1997). Coronary risk factors and plaque morphology in men with coronary disease who died suddenly. N Engl J Med.

[7] Ueda Y, Ogasawara N, Matsuo K, Hirotani S, Kashiwase K, Hirata A (2010). Acute Coronary Syndrome:Insight From Angioscopy. Circ J.

[8] Erne P, Radovanovic D, Seifert B, Bertel O, Urban P (2015). Outcome of patients admitted with acute coronary syndrome on palliative treatment:insights from the nationwide AMIS Plus Registry 1997–2014. BMJ Open.

[9] Alvaro Avezum A, Makdisse M, Spencer F, Gore JM, Fox KAA, Montalescot G (2005). Impact of age on management and outcome of acute coronary syndrome:Observations from the global registry of acute coronary events (GRACE). AHJ.

[10] Roffi M, Patrono P, Collet JP, Mueller C, Valgimigli M, Andreotti F (2016). 2015 ESC Guidelines for the management of acute coronary syndromes in patients presenting without persistent ST-segment elevation:Task Force for the Management of Acute Coronary Syndromes in Patients Presenting without Persistent ST-Segment Elevation of the European Society of Cardiology (ESC). Eur Heart J.

[11] Alfredsson J, Stenestrand U, Wallentin L, Swahn E (2007). Gender differences in management and outcome in Non-ST-Elevation Acute Coronary Syndrome. Heart.

[12] Fanaroff AC, Rymer JA, Goldstein SA (2015). Acute Coronary Syndrome. JAMA.

[13] Radovanovic D, Erne P, Urban P, Bertel O, Rickli H, Gaspoz JM (2007). Gender differences in management and outcomes in patients with acute coronary syndromes:results on 20,290 patients from the AMIS Plus Registry. Heart.

[14] Duan JG, Chen XY, Wang L, Lau A, Wong A, Thomas GN (2015). Sex Differences in Epidemiology and Risk Factors of Acute Coronary Syndrome in Chinese Patients with Type 2 Diabetes:A Long-Term Prospective Cohort Study. PLoS ONE.

[15] Yang B, Fan S, Zhi X, Wang Y, Wang Y, Zheng Q (2015). Prevalence of Hyperhomocysteinemia in China:A Systematic Review and Meta-Analysis. Nutrients.

[16] Shah H, Jan MU, Altaf A, Salahudin M (2018). Correlation of hyper-homocysteinemia with coronary artery disease in absence of conventional risk factors among young adults. J Saudi Heart Assoc.

[17] (2018). Pakistan Demographics Profile.

[18] Rajhans R, Narayanan M (2017). Assessment of arrhythmias in 50 patients of ST-elevation myocardial infarction after thrombolysis:a 24 hour Holter study. Int J Adv Med.

[19] Shah H, Salahuddin M, Altaf A (2016). Association of Left Ventricular Ejection Fraction and gender differences in acute decompensated heart failure patients. J Rehman Med Inst.

[20] Kawut SM, Lima JA, Barr RG, Chahal H, Jain A, Tandri H (2011). Sex and race differences in right ventricular structure and function. The multi-ethnic study of atherosclerosis – Right ventricular study. Circulation.

[21] Ijaz A, Ijaz A, Saad AA, Zafar S, Safdar S, Rafique S (2017). Renal Dysfunction;In Patients With Acute Coronary Syndrome (ACS) At A Tertiary Care Hospital. Prof Med J.

[22] Udell JA, Koh M, Qiu F, Austin PC, Wijeysundera HC, Bagai A (2017). Outcomes of Women and Men With Acute Coronary Syndrome Treated With and Without Percutaneous Coronary Revascularization. JAHA.

[23] Torabi A, Cleland JG, Rigby AS, Sherwi N (2014). Development and course of heart failure after a myocardial infarction in younger and older people. J Geriatr Cardiol.

[24] Mansouri A, Tasharoie S, Javidee S, Kargar M, Taghizadeh-ghehi M, Hadjibabaie M (2014). Streptokinase Adverse Reactions:A Review of Iranian Literature. J Pharma Care.

